# Operative fixation of an intra-articular scapula fracture from household appliance electrocution: a case report

**DOI:** 10.1016/j.xrrt.2025.08.007

**Published:** 2025-08-18

**Authors:** Michael B.C. Newton, Charlotte Allen

**Affiliations:** Department of Orthopaedics, Te Whatu Ora - Counties Manukau, Auckland, New Zealand

**Keywords:** Scapula, Fracture, Electric injuries (MeSH), Operative procedure (MeSH), Glenoid fracture, Open reduction and internal fixation, Low-voltage electrocution injury, Household electrocution

Scapula fractures are rare injuries, comprising less than 1% of fractures presenting to the emergency department.^11^ While glenohumeral dislocations have been associated with abnormal electrical stimulation of the shoulder girdle muscles, there is a paucity of reports describing scapular fracture from this mechanism. We present a unique case of glenoid and scapular body fractures following exposure to a household electrical supply, requiring surgical fixation. This report provides a comprehensive review of the mechanism of injury, details the operative strategy, and summarizes initial outcomes for this rare and complex presentation.

## Case report

A healthy 27-year-old gentleman presented to hospital after suffering an electrocution injury from an electric frypan. While trying to insert the cable into the 1800-watt pan connected to a home socket, (New Zealand; 230-240V, 50 Hz AC), his right little finger was caught against the exposed metal terminals. The electric shock radiated through his arms and chest, causing him to fall. He denied any direct impact to his right side/shoulder. He experienced bilateral upper limb tetany for several seconds after the shock, however, did not lose consciousness.

**Figure 1:**
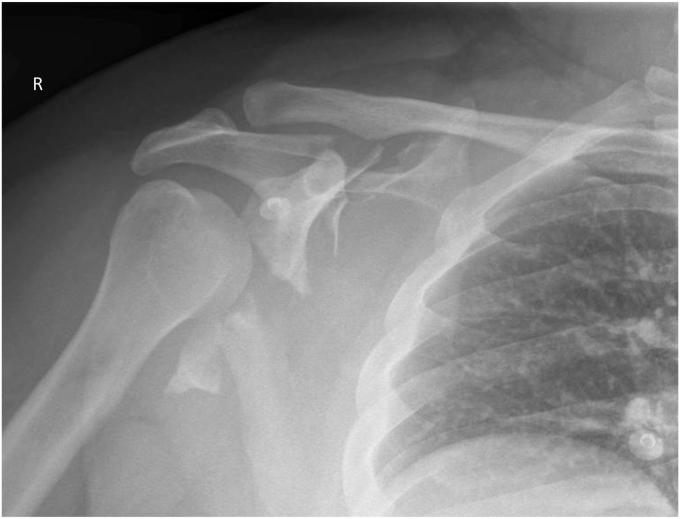
Initial plain radiographs.

**Figure 2:**
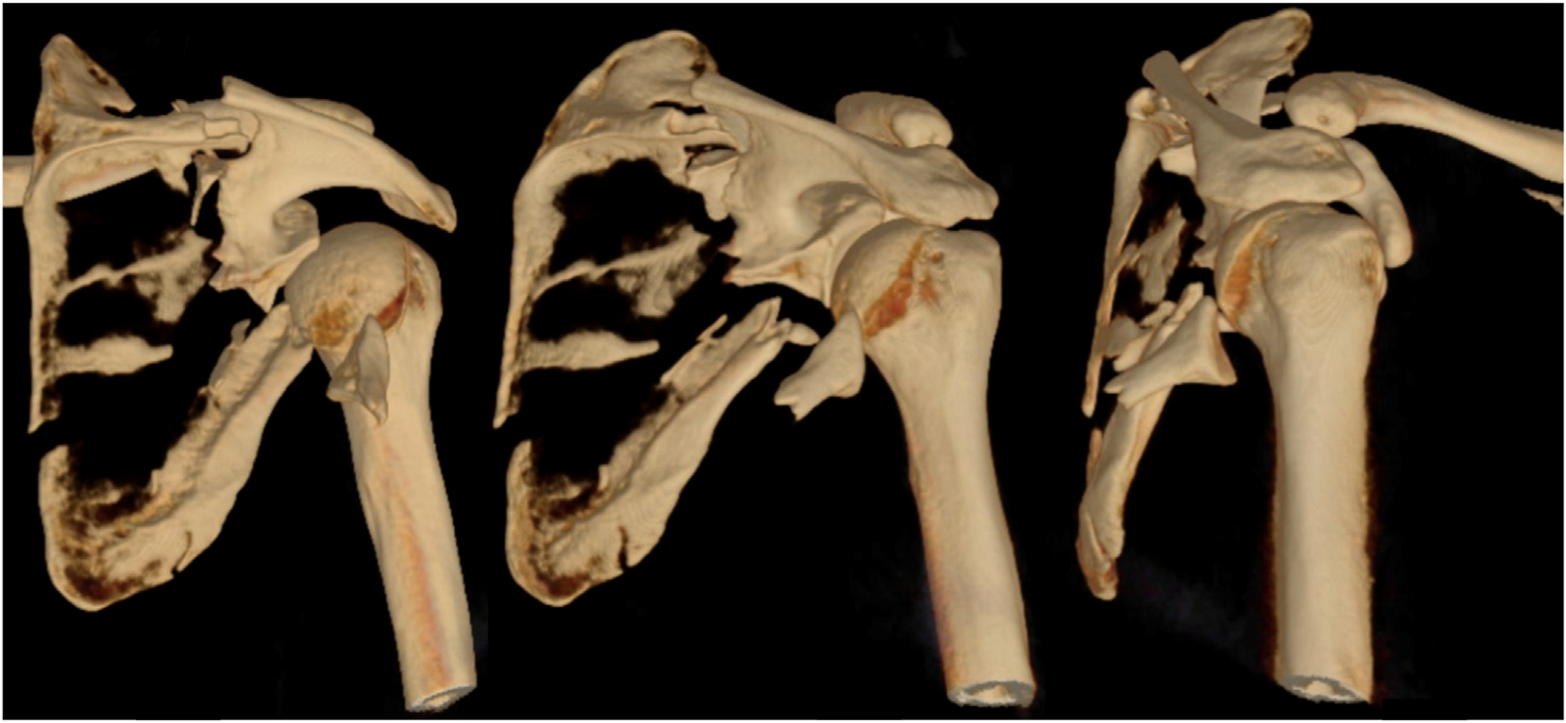
CT scan with 3-dimensional reconstruction. *CT*, computed tomography.

On admission, his complaints were intermittent chest pain and palpitations, plus right shoulder pain and immobility. He was examined according to Emergency Management of Severe Burns protocol.^15^ Significant findings included a 3 mm circular entry burn at the right little finger. No exit point was found. His bilateral upper limbs had no clinical deformity, but he had trouble mobilizing his right shoulder. Neurovascular examination was intact and compartments soft. Continuous cardiac monitoring was initiated, showing sinus rhythm, and blood tests displayed a rising creatinine kinase to 2,324 units/liter (NR 60-220 U/L), but no other abnormality. Plain films of his right shoulder showed a comminuted fracture, with a transverse split of his glenoid; displaced anteriorly and inferiorly (Fig. 1). His humeral head remained relatively enlocated, and pattern was consistent with an Arbeitsgemeinschaft für Osteosynthesefragen (AO) 14B:2 fracture shown by a subsequent computed tomography (Fig. 2). He was admitted to the ward for telemetric monitoring and operative planning.

**Figure 3:**
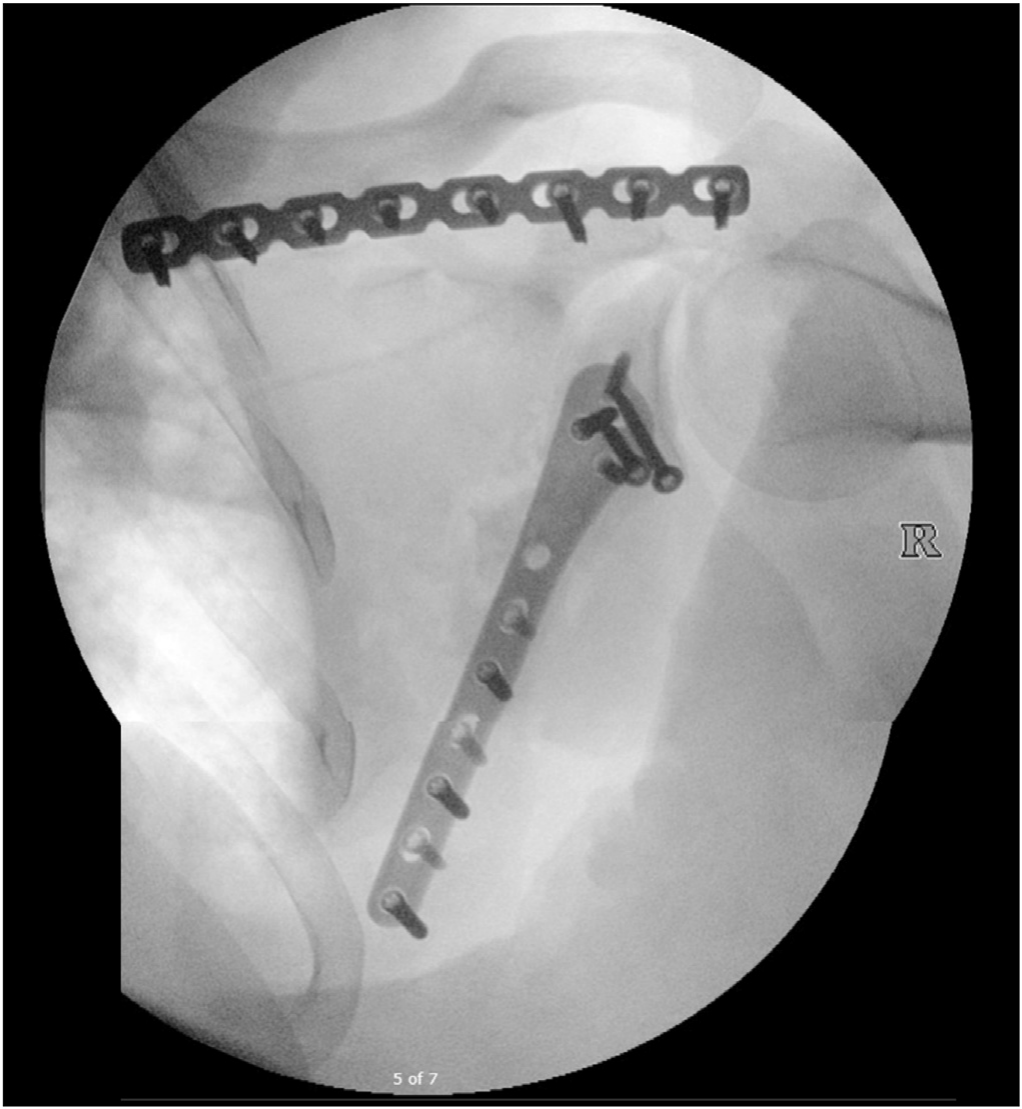
Intraoperative fixation films.

Operative fixation was completed due to the fracture displacement of the glenoid, aligning with the AO principle of anatomic fixation with absolute stability for intra-articular fractures.^2^ Under general anesthesia, the patient was placed prone to facilitate access to the fracture through a modified Judet approach. An incision following the scapula's spine and medial border was made down to fascia.^9^ The posterior deltoid was reflected encountering the fractured scapula spine which was reduced and plated using a Synthes pelvic recon plate. The interval between infraspinatus and teres minor was utilized to reduce the lateral border and inferior glenoid piece with lag screws before placement of an Accumed lateral scapula plate (Hillsboro, OR, USA). Final X-rays were taken showing no screws in the joint (Fig. 3). After copious irrigation using pulse lavage 1 gram of vancomycin powder was left in the deep wound, and closure was completed in a layered fashion.

The patient was managed postoperatively in a broad-arm sling, non–weight-bearing for 4 weeks before starting pendular exercises, graduated range of motion, then weight bearing. He regained abduction to 130° at his 6-week follow-up, his wounds healed well, and he did not suffer any lasting neurological sequelae. Further reviews at 3 and 6 months showed return to normal active range of motion, and he has returned to full-time work. There is no plan for reoperation for plate removal; however, he has been counseled regarding post-traumatic shoulder arthritis.

## Discussion

This case report emphasizes the importance of screening for musculoskeletal injuries in the setting of electrocution, even in that from household supply. While rare, the usual association is a posterior shoulder dislocation.^10^ These have been described in the setting of epilepsy, cardiopulmonary resuscitation, or electroconvulsive therapy prior to the routine administration of muscle relaxants.^12^ Pain, immobility and neurological deficit are expected after an electric shock. Therefore, one must have high suspicion, and examine the affected limb carefully to exclude fracture, often characterized by marked shoulder drooping or ptosis on the injured side. The Australian and New Zealand Burn Association recommend chest x-ray as standard workup for burns, of which electrocution is a subtype. There is potential to incidentally image a fractured scapula, however, overlay of the ribcage and lungs, alongside a low suspicion may make this less apparent.^15^ Furthermore, this mechanism is unlikely to initiate a trauma call or computed tomography trauma protocol. Dedicated shoulder girdle X-rays, particularly a scapular Y view, should be completed if injury is suspected.

A review of the literature found no other reported cases of an intra-articular fracture of the glenoid and scapular body caused by electrocution. The review suggests that scapular body fractures from electrocutions are primarily associated with low voltages (Table I). All 9 report low-voltage electrocutions causing fractures to the scapular body, with Beswick, Chen and Wencheng mentioning the absence of a fall or direct trauma, as in our case. As duration of shock is subjective it is difficult to assess if prolonged time was correlated with bilateral vs. unilateral fractures. Chen was unique in requiring operative management due to displacement of the extra-articular lateral body; however, that fracture was extra-articular, and technique not described.^3^ There is merit in fixation of some extra-articular fractures where outcomes may be improved with surgery, such as involvement of the scapular neck, increased polar angle or displaced rib fractures.^11^ Operative management was clearly required in this case due to displacement of the articular surface. It has been well-fixed with excellent function at 6 months postoperatively.

**Table I:** Literature review

Author	Injury	Voltage (V)/frequency (Hz)	Management
Chen, H. (2024)^3^	Single scapular body	Not stated	Operative
Huang, W (2010)^6^	Single scapular body	110/60	Nonoperative
Rana, M. (2006)^10^	Single scapular body	Household	Nonoperative
John, BS. (2004)^7^	Bilateral scapular body	240/not stated	Not stated
Kotak, BP. (2000)^8^	Bilateral scapular body	240/60	Nonoperative
Dumas, JL. (1992)^4^	Bilateral scapular body	220/50	Nonoperative
Simon, JP. (1991)^13^	Single scapular body	Household	Nonoperative
Henneking, K (1984)^5^	1. Single scapular body 2. Bilateral scapular bodies	1. High-voltage power line 2. Household	Nonoperative in both instances
Beswick, DR. (1982)^1^	Bilateral scapular body	440/60	Nonoperative
Tarquinio, T. (1979)^14^	Bilateral scapular body	440/60	Nonoperative

Electrical injuries can cause significant morbidity, often with damage that is not immediately evident on external examination.^15^ High-voltage, direct current induces a single, forceful contraction, often ejecting the individual. These can cause both flash burns, deep tissue injuries from the shock as well as from being thrown/falling. Skin and bone have higher resistance than muscle and nerves, which in high-voltage electrocutions can cause heat-induced fractures and severe myonecrosis from heat dissipation from bones (in accordance with Joule's law).^15^^,^^10^ In the instance of low voltages, Ohm's law is more apparent; high resistance of skin limits superficial damage; current preferentially flows internally and masks the true extent of injury. Simon et al report on a case where a scapular fracture wasn't discovered for 3 weeks after injury.^13^ In alternating current circuits, tetanic muscle contractions can also prolong exposure by preventing the individual releasing the source, as reported in multiple cases.^15^^,^^3^^,^^10^ As with the other reports of this injury, we posit that sustained tetanic contraction of the shoulder girdle musculature generated sufficient force to drive the humeral head through the glenoid and scapula, resulting in a comminuted fracture. This highlights the need for careful musculoskeletal assessment in electrocution cases, even at household voltages.

## Conclusion

Scapular fractures resulting from household electrocution are exceptionally rare and require high clinical suspicion for timely diagnosis. This case highlights the importance of thorough musculoskeletal assessment and supports encouraging outcomes from operative fixation when there is significant articular displacement.

## Disclaimers

Funding: No funding was disclosed by the authors.

Conflicts of interest: The authors, their immediate families, and any research foundations with which they are affiliated have not received any financial payments or other benefits from any commercial entity related to the subject of this article.

Patient consent: Obtained.
